# Supplementary material for: Diversity and Genetic Structure of Dioon holmgrenii (Cycadales: Zamiaceae) in the Mexican Pacific Coast Biogeographic Province: Implications for Conservation
Source: Plants (Basel). 2021 Oct 21;10(11):2250. doi: 10.3390/plants10112250 (PMC8623071; doi:10.3390/plants10112250)
Supplement: Supplementary file 1 [file plants-10-02250-s001.zip › plants-1370687-supplementary.pdf]

Article

Diversity and genetic structure of *Dioon holmgrenii* (Cycadales: Zamiaceae) in the Mexican Pacific Coast biogoegraphic province: implications for conservation

Table S1. Nei genetic distances (above the diagonal) and geographic distances (Km, below the diagonal) between nine populations of *Dioon holmgrenii*.

**Populations**	**Río Leche**	**La Lima**	**Ocotlán**	**Rancho Viejo**	**Rancho el Limón**	**Cerro Antiguo**	**Cieneguilla**	**Cerro Caballo**	**San Bartolomé**
Río Leche		0.206	0.516	2.381	4.036	1.629	2.989	1.507	1.357
La Lima	4.807		0.398	1.777	2.650	1.639	2.281	1.493	1.427
Ocotlán	28.983	27.482		0.955	1.659	1.763	1.431	1.513	1.371
Rancho Viejo	66.220	63.730	38.910		0.300	0.941	0.121	0.603	0.783
Rancho el Limón	104.651	101.837	7.770	33.445		0.836	0.067	0.620	0.825
Cerro Antiguo	115.494	112.700	87.924	45.380	12.879		0.804	0.205	0.130
Cieneguilla	120.860	117.818	93.456	54.441	18.095	5.078		0.527	0.731
Cerro Caballo	121.526	118.867	95.000	55.558	17.823	2.672	4.782		0.066
San Bartolomé	135.340	132.610	107.999	69.104	31.467	19.844	15.337	13.555	

Table S2. Fst values (below the diagonal) and probability (above the diagonal) between pairs of *Dioon holmgrenii* populations.

Populations	Río Leche	La Lima	Ocotlán	Rancho Viejo	Rancho El Limón	Cerro Antiguo	Cieneguilla	Cerro Caballo	San Bartolomé
Río Leche		0.001	0.001	0.001	0.001	0.001	0.001	0.001	0.001
La Lima	0.045		0.001	0.001	0.001	0.001	0.001	0.001	0.001
Ocotlán	0.063	0.069		0.001	0.001	0.001	0.001	0.001	0.001
Rancho Viejo	0.230	0.251	0.159		0.001	0.001	0.001	0.001	0.001
Rancho El Limón	0.241	0.269	0.192	0.120		0.001	0.001	0.001	0.001
Cerro Antiguo	0.171	0.203	0.159	0.206	0.193		0.001	0.001	0.001
Cieneguilla	0.229	0.256	0.178	0.055	0.035	0.184		0.001	0.001
Cerro Caballo	0.179	0.209	0.163	0.171	0.173	0.066	0.153		0.002
San Bartolomé	0.174	0.208	0.159	0.200	0.205	0.048	0.187	0.024	

Table S3. Characteristics of the SSR microsatellite starters for nine *Dioon holmgrenii* populations. UR: repeating unit, NA: number of alleles, Size: size, EF: fluorescent label, TA: alignment temperature, F: forward primer, R: reverse primer.

Group	Locus[36, 53]	Primer sequenses (5´-3´)	Type repeat	Number of alleles	Size (pb)	Dye (forward primer)	Annealing temperature (°C)
1	Ed3	F: GCATGAGGAGCTTGTTCCGT	$(CT)_{19}$	7	88-98	6-FAM	58.1
		R: CTGTGAACTCCTGAAAGCATC					
1	Cap5	F: CACTACCACCCCTATACCAC	$(CT)_{23}$	7	162-173	HEX	58.1
		R: GACTTGAGCTTGTCTTTGTTG					
2	Ed5	F: AGGCATAAATGGCTAAGCATAC	$(AG)_{16}$	12	149-167	HEX	57.3
		R: GCATTTCTAGTGGACAAACCAG					
2	Ed6	F: ATGCAGATGAAACACACCC	$(TGG)_{8}$	5	119-154	ROX	57.3
		R: TCCTAACCATCCATCACTACC					
3	1660	F: GGTGCTGAAGAGGAAGAAGAA	$(GAA)_{16}$	14	171-248	6-FAM	58.9
		R: AGGGGAGAAGACATAACAAAGT					
3	Zam29	F: GACTTCCTACCTAAAGATGCTA	$(AGT)_{15}$	13	161-247	HEX	58.9
		R: TCCTGCTTACTTTAGATGCT					

*
